# The unseen symptom: A longitudinal qualitative interview study exploring mobility loss in people with advanced cancer

**DOI:** 10.1177/02692163251400115

**Published:** 2025-12-26

**Authors:** Carmine Petrasso, Lisa Jane Brighton, Matthew Maddocks, Joanne Bayly

**Affiliations:** 1 Cicely Saunders Institute of Palliative Care, Policy and Rehabilitation, King’s College London, England, UK

**Keywords:** mobility, neoplasms, palliative care, rehabilitation

## Abstract

**Background:**

Mobility loss is a common symptom in people with advanced cancer and is associated with reduced function and quality of life. While its physical consequences are well documented, less is known about how individuals and their families experience and adapt to these changes over time.

**Aim:**

To explore how individuals with advanced cancer and their caregivers experience and respond to mobility loss.

**Design:**

A qualitative longitudinal study using Interpretative Phenomenological Analysis. Participants completed two semi-structured interviews, with follow-ups prompted by changes in mobility.

**Setting/participants:**

Twelve participants (10 people with advanced cancer, 2 caregivers) were recruited from hospital services, hospices, and cancer charities across England. Interviews were conducted 6–24 weeks apart in person, online, or via telephone.

**Results:**

Four interrelated themes were identified: (i) *Fractured embodiment: Loss of the mobile self*; (ii) *Mobility as a cornerstone: Disruption to the fabric of daily life*; (iii) *Resisting and reframing loss of mobility*; and (iv) *Mobility loss: The unseen symptom*. Mobility loss disrupted identity, agency, and participation, and led to reconfigured family roles. Coping with mobility change was dynamic and involved reframing expectations and negotiating autonomy. Participants often faced delayed or absent support, with unmet mobility needs frequently overlooked by clinicians.

**Conclusions:**

Mobility loss in advanced cancer is not only a symptom of disease, but also a personal and relational disruption. Recognising it as a multidimensional experience underscores the need for holistic, anticipatory care that supports both physical functioning and the emotional realities of serious illness.

**What is already known about the topic?**

- Mobility loss is a common symptom in advanced cancer, associated with poorer function and quality of life.
- The emotional and relational impacts of mobility loss are underexplored in qualitative cancer research.

**What this paper adds?**

- This study shows that mobility loss disrupts identity, erodes social roles, and alters family dynamics in people with advanced cancer.
- Coping with mobility loss is an evolving process involving emotional reframing, practical adaptation, and negotiating autonomy.
- Mobility needs are frequently overlooked by clinicians, with participants often independently sourcing assistive equipment and support.

**Implications for practice, theory or policy**

- Mobility loss should be routinely identified in advanced cancer and addressed early through rehabilitation and assistive interventions.
- Recognising the emotional meaning of mobility can guide more person-centred discussions and reduce stigma around assistive product use.

## Introduction

Cancer remains a leading cause of death, with advanced cancer associated with high symptom burden for patients and caregivers.^1^ Mobility loss is a significant yet under-recognised symptom of advanced cancer that can affect daily activities, emotional wellbeing, and participation.^2,3^

Mobility is the ability to move within different environments and encompasses activities such as walking, transferring, and navigating physical spaces.^4^ Mobility loss represents a decline in these abilities, ranging from reduced walking distance to complete loss of independent movement.^5^ In advanced cancer, mobility loss may arise from a combination of factors relating to disease progression and treatment-related side effects.^6^ This rate of decline may be rapid, but for many it is gradual and progressive, reflecting the cumulative impact of interrelated processes such as fatigue, cachexia, pain, and breathlessness.^6^ These processes interact over time, creating a cycle of decline.^7^ Worsening symptoms contribute to loss of mobility, and reduced mobility further exacerbates physical deconditioning compounding symptom burden.^6,8^ These limitations subsequently affect activities of daily living and often result in increased reliance on caregivers.^9,10^

Despite its importance, mobility loss is rarely prioritised in cancer care compared with symptoms such as pain, breathlessness, or fatigue.^11^ Yet, mobility is an important determinant of independence, dignity, and quality of life, and its loss increases caregiver strain and role burden.^10,11^ Quantitative studies have shown that declines in physical function are associated with lower quality of life and heightened psychological distress in advanced cancer.^11–14^ However, less is known about how these changes are experienced by individuals and caregivers in everyday life. While previous qualitative work has examined the effects of sudden mobility loss following metastatic spinal cord compression,^15^ or explored mobility in patients with pre-existing impairments,^16^ these studies focus on specific subgroups and offer limited insight into the broader experiences of people with advanced cancer, for whom mobility decline is often more gradual and progressive.

There is a need for research that explores how mobility loss disrupts people’s lives and how they adapt over time. Exploring these experiences longitudinally, here defined as repeat interviews with the same participants over time, enables examination of evolving trajectories of disruption and adaptation.^17,18^ Such designs capture not only the immediate impact of mobility loss but also how coping strategies, role negotiations, and support needs shift as illness advances.^18^ A deeper understanding of these experiences may help identify unmet needs, inform more responsive support, and guide the development of tailored interventions. Therefore, this study aimed to (i) explore the impact of mobility changes on individuals with advanced cancer and their caregivers, and (ii) examine the coping strategies used by patients and caregivers in response to mobility changes.

## Methods

### Design

This qualitative longitudinal study used interpretative phenomenology and involved two semi-structured interviews to examine mobility changes over time. The study is reported in line with the COREQ guidelines.^19^

### Population

Eligible participants were adults (⩾ 18 years) living in England with a self-reported stage III–IV cancer diagnosis, and/or their informal caregivers. Caregivers were defined as family members or friends providing unpaid support. Where multiple caregivers were present, the individual most involved in daily support was invited to participate, as identified by the patient. Participants were excluded if they were unable to provide informed consent or lacked sufficient English language proficiency to take part in an interview.

### Setting

Participants were based in community and clinical settings across England. Recruitment sites included hospitals providing oncology and palliative care, hospices, and cancer charities. Interviews were conducted either in participants’ homes, in clinical settings, or online, depending on participant preference.

### Sampling

The sample size was guided by the concept of “information power,” whereby smaller samples are appropriate when cases are rich, focussed, and aligned with the study aim.^20^ In line with interpretative phenomenological analysis’ idiographic approach, the focus was on depth rather than breadth, enabling detailed exploration of meaning-making in relation to mobility changes.^21^ Recruitment continued until data offered sufficient richness to address the study objectives, with recurring themes evident across cases. While recruitment was partly constrained by the doctoral time frame, patient interviews were sufficiently rich to address the primary research objectives. The smaller number of caregiver participants meant their perspectives were used primarily to enrich understanding of relational dynamics rather than to represent the full complexity of caregiving experience.

### Recruitment

Recruitment materials were distributed through posters in hospitals, hospices, and cancer charities, as well as via social media. Interested individuals contacted the research team directly and were screened for eligibility. Both patients and caregivers could enrol independently or as part of a dyad.

### Data collection

Participants completed a demographic questionnaire and took part in two semi-structured interviews. All interviews were conducted by CP (male doctoral researcher, qualified physiotherapist and nurse with qualitative interview training). The first interview, at enrolment, explored mobility changes since diagnosis. The second interview took place when participants reported a change in their mobility. To review for any changes, participants were contacted every 3–4 weeks by telephone.

Interviews were conducted in person or online, based on participant preference, and took place either individually or, where patients and caregivers enrolled as a dyad, together, using a flexible topic guide co-developed with patient and public involvement members (Supplemental File 1). The guide supported participant-led discussions and was refined iteratively, informed by early interpretations and analytical engagement with the data across interviews. All interviews were audio-recorded, transcribed verbatim, and supplemented with field notes capturing contextual details, non-verbal cues, and researcher reflections.

### Data analysis

We used Interpretative Phenomenological Analysis (IPA) within a critical realist framework, which recognises that while a material reality exists, such as illness or physical decline, our understanding of it is shaped by individual interpretation and influenced by social, cultural, and personal contexts.^22^ IPA was chosen for its idiographic focus and its emphasis on how participants make sense of lived experiences over time, providing a detailed account of meaning-making within these broader frameworks.^21,23^ The study did not begin with predefined theories; instead, theoretical frameworks were engaged inductively during analysis to deepen interpretation of the findings.

Analysis was conducted by CP, LB, and JB, following an iterative, multi-stage process. Transcripts were read and re-read to immerse in participants’ accounts, with descriptive, linguistic, and conceptual notes recorded. Data contributing to initial insights were highlighted, underlined, and annotated by hand to trace the development of meaning within interviews.^21^ Themes were then developed through a process of organising quotes and concepts using sticky notes and visual mapping techniques to explore connections within and across cases. A coding tree was created to document sub-themes, interpretative comments and illustrative quotations. Themes were analysed for patterns across cases while preserving individual narratives.

Reflexivity was maintained through ongoing team discussions, where researchers critically examined their own perspectives and potential biases. These conversations ensured that the analysis remained participant-centred and interpretative.^24^ Recognising the limited representation of caregivers, and the absence of minoritised ethnic participants, a supplementary workshop was conducted with three bereaved caregivers from minoritised communities who had cared for someone with advanced cancer. This served two purposes: to gain additional insight into caregiving experiences, and to reflect on the cultural relevance of the findings. Strategies to ensure trustworthiness were applied in line with Lincoln and Guba’s criteria; these are summarised in Table 1.^25^

**Table 1.** Strategies used to ensure trustworthiness.

Criterion	How it was fulfilled
Credibility	Supported through iterative engagement with transcripts, repeated reading, and team discussions to check interpretations against the data. Credibility was further strengthened by patient and public involvement in co-developing the interview guide, ensuring questions reflected participant concerns, and by using illustrative quotations to ground interpretations in participants’ own words.
Dependability	Ensured by detailed documentation of the analytic process, including coding notes, visual mapping, development of a coding tree, and records of how themes were refined over time.
Confirmability	Maintained through reflexive dialogue within the research team, where assumptions were critically examined and alternative interpretations discussed. Field notes were also used to capture contextual detail and researcher reflections during data collection.
Transferability	Supported through detailed description of participants’ accounts and study context to allow readers to consider relevance to other settings. A supplementary workshop with bereaved caregivers from minoritised ethnic backgrounds also enabled reflection on the resonance of findings beyond the primary sample.

### Ethical issues

Ethical approval was obtained from Southwest Frenchay Research Ethics Committee (24/SW/0052) in April 2024. Participants provided written consent, were aware that the study formed part of a doctoral project, could withdraw at any time without consequence, and were offered appropriate support resources if needed.

## Results

Twelve participants (10 patients, 2 carers – Table 2) completed 2 interviews each, totalling 20 interviews between July 2024 and April 2025, including 4 dyadic interviews. Eleven interviews were conducted in person (10 at home, 1 at a hospice), 8 online, and 1 by telephone due to rapid decline. First interviews lasted 50–120 min; follow-ups 15–90 min, mean time between interviews was 16 weeks (range: 6–24 weeks). To ensure anonymity, pseudonyms have been assigned to all participants. Results are presented by theme, and individual stories are provided as short biographies (Supplemental File 2). The key themes and sub-themes are presented in Table 3. Drawing on Carel’s phenomenology of illness, the first three themes reflect participants’ lived, first-person experience of their changing mobility (see Table 3), while the fourth theme; “Mobility loss: The unseen symptom,” captures how their mobility was perceived, and often overlooked, by others.^26^ The fourth theme therefore acts as a bridge between the subjective disruption of embodiment and the social and clinical recognition required to access support.

**Table 2.** Participant demographics.

Participant characteristic	Total
Sex	
Female	7
Male	5
Age (Years)	
Range	50–81
Mean	71
Marital status	
Married	10
Widowed	1
Single	1
Occupation	
Retired	7
Medically retired	3
Self-employed	1
Ethnicity	
White British	12
Cancer site	
Breast	4
Colon	2
Multiple myeloma	1
Oesophageal	1
Prostate	1
Renal	1

**Table 3.** Themes and sub-themes.

Theme	Sub-themes	Interpretation	Illustrative quotations
Fractured embodiment: Loss of the mobile self	Grieving the pre-illness identity	Participants described mobility loss as a dissociation between their past and present selves, creating a sense of discontinuity in how they recognised themselves.	“*It’s getting me down. I can’t walk the way I used to. . .I just can’t walk at all sometimes*” – Annie “*My life is in the rearview mirror now, its all gone, that’s it. I can’t go back*” – Daniel
	Dissociation from the body	Alongside grief for the former self, some described a disconnect between body and identity, describing their body as alien or untrustworthy.	“*It’s like my body has betrayed me. I don’t even feel like I’m living my own life anymore*” – Daniel “*It’s just, I can’t control my legs, they feel like they’re just flopping about*” – Philip “*Now it feels like I have to ask permission from my own body to move*” – Charles
Mobility as a cornerstone: Disruption to the fabric of daily life	Eroding participation	With mobility loss, everyday activities and social connections diminished, creating a “shrinking world” that limited spontaneity and reinforced isolation.	“*I can’t go out and do the things I used to, everything’s just so difficult*” – Annie “*I do try to still meet up with friends, but its difficult because they’re all talking about all the things they’re doing, the grandchildren, and all the stuff they’re getting up to and I just sit there and can’t say nothing. I’m not going anywhere, not doing anything*” – Daniel “*I used to go for long walks with my wife, but now I can’t even manage a short walk*” – Charles
	Redefining roles in relationships	The erosion of independence reshaped family roles. Alongside eroding participation, participants described guilt and frustration at relying on others, while caregivers spoke of renegotiating responsibilities within relationships.	“*I’m so dependent on my kids now. I feel like I’m taking away their lives*” – Sharon “*I feel less like a wife and more like a nurse again. I can handle him being ill, but the immobility has been really challenging. It came on so suddenly*” – Patricia
Resisting and reframing loss of mobility	Negotiating autonomy through adaptation	Participants used coping strategies that reframed their situation, adjusting expectations and focussing on what they could still achieve.	“*Some days I want to give up, but I just remind myself that I’m still here, and I can still try*” – Lorraine “*I just try to get on with it. If I let it take over, I won’t be able to cope*” – Philip
	Evolving interdependence through mobility devices	Acceptance of mobility devices was often a turning point. Devices were reframed from symbols of decline to tools that supported safety, independence, and participation.	“*I use a frame to get around the house, it’s frustrating, but it’s better than nothing*” – Annie “*The wheelchair is a godsend when I need it. It’s not what I wanted, but it’s what I need*” – Lorraine “*Without the scooter, I couldn’t go anywhere. . .it helps me feel a little more normal*” – Peter
Mobility loss: The unseen symptom	Missed opportunities for support	Despite the centrality of mobility to daily life, participants felt it was rarely prioritised in care. Support was often delayed or only offered after crisis points.	“*Nobody ever asked me about walking. It wasn’t on their radar. They were focussed on the cancer, but I was struggling just to get to the bathroom*” – Kristen “*I don’t think anyone [oncologists] has ever asked about my walking*” – Elizabeth “*We only got help once when we hit breaking point. Before that, we were on our own*” – Matthew
	Stigma, ageism and illness assumptions	Some participants described their mobility concerns being normalised or dismissed as part of ageing or advanced illness, reinforcing the sense that mobility needs were overlooked.	“*When I mentioned my walking was getting worse, I was told, ‘Well you’re not getting any younger.’ But that’s not the point, I know my body, and this isn’t just age*” – Elizabeth “*It’s almost like if you’re older and ill, people expect you to be immobile. So, they don’t see it as something to fix or help with*” – Claire

### Fractured embodiment: Loss of the mobile self

#### Grieving the pre-illness identity

Participants described a disruption to identity following mobility loss. Many saw their lives as split between a “before” and “after,” with illness marking the transition from a previously active independent self, to one they no longer recognised. This discourse was experienced not only as a physical decline, but as a disruption to how participants understood and related to themselves, often describing a form of grief for their former selves:

“*I used to be the strong one in the family, always on my feet and doing everything. Now I look in the mirror and I barely recognise myself*” – Philip.

For many, walking held a symbolic significance: it represented independence, freedom, and connection to the world. Mobility loss was not only a practical limitation, but it impacted self-definition. In some cases, this sense of loss affected participants’ inner worlds, shaping their thoughts and their dreams:

“*I sometimes find myself dreaming about erm, even about walking, I even dream about running sometimes. In my dream I see myself running and sort of stuff like that. Possibly because there’s nothing in the present, even my dreams are living in the past now*” – Daniel.

The loss of mobility also affected caregivers’ sense of identity. The shift from partner to carer was experienced not only as a change in responsibility, but as a loss of relational identity:

“*I feel less like a wife and more like a nurse again. I can handle him being ill, but the immobility has been really challenging. It came on so suddenly*” – Patricia.

#### Dissociation from the body

Many participants described a growing sense of estrangement from their own bodies. Once reliable and integral, their body became something unfamiliar, failing to cooperate, and no longer reflecting the self that participants used to recognise:

“*I can’t even get out of bed without help now. I used to jump out of bed, ready to start the day. Now it feels like I have to ask permission from my own body to move. It doesn’t feel like me anymore, it’s just a shell of what I used to be. It’s like I’m losing control over my own body, and I’m scared of what’s next. Every time I look at my hands or legs, they seem foreign to me*” – Charles.

### Mobility as a cornerstone: Disruption to the fabric of daily life

#### Eroding participation

Mobility loss was described not only as a symptom, but as a fundamental disruption to the way participants lived and found meaning in their everyday worlds. It limited spontaneity, social contact, and turned routine outings into complex tasks. Many described a “shrinking world,” where everyday activities became difficult or impossible:

“*My world has definitely gotten a lot smaller. . . I had to cancel a dentist appointment as I’m housebound. It’s a word that I hadn’t thought of and didn’t want to use, but that’s what I am now. Housebound.*” – Annie.

Some participants showed resourcefulness in finding alternative ways to stay socially engaged by replacing face-to-face activities with remote or more sedentary ones. Despite this, participants often described that the consequence of social withdrawal was often a sense of boredom, loneliness, or loss of purpose:

“*All I’ve got now is TV programmes. I just spend my day in the chair and watch the news, and I almost know it off by heart. . . I’m just getting through the day really*” – Charles.

The loss of mobility was closely tied to a loss of agency, with some participants describing feelings of being left behind, and perceiving oneself as receding into the past:

“*It just seems like everything is in the past now y’know? My life is in the rearview mirror now, its all gone, that’s it. I can’t go back*” – Daniel.

#### Redefining roles in relationships

As mobility changed, participants frequently spoke about being unable to fulfil their usual roles within the family and community: roles that had previously defined their purpose and social identity. Everyday activities, including simple household tasks became difficult. This enforced dependency was described with feelings of frustration and guilt, with participants acknowledging the strain placed on family. They worried about being a “burden” and losing the reciprocal role they once held in relationships:

“*When he [husband] comes in [from work], he has to do everything. And I feel so guilty. It just doesn’t seem fair on him. He’s been to work all day. . . I wish I could do more to make life easier*” – Annie.

Caregivers described their own lives being altered. Some framed mobility loss as a relational challenge, not just personal. Couples had to renegotiate shared routines and give up activities they once enjoyed:

“*I think the difficulty with her [Claire’s] walking has disabled us a couple. We were both pretty upbeat when this first started, but now our universe is collapsing. We just can’t run away and escape on a nice cruise, it would be impossible and it’s just constant. . . Life isn’t life anymore, it’s just an existence*” – Matthew.

### Resisting and reframing loss of mobility

#### Negotiating autonomy through adaptation

Although mobility loss posed emotional and relational challenges, participants demonstrated a variety of strategies for coping and adapting. These were not static responses, but evolving processes shaped by disease progression, psychological resilience, and available support. Many participants described adjusting expectations and finding creative ways to preserve a sense of autonomy or purpose, with several participants focussing on what they could still do rather than on what was lost:

“*I’ve had to learn to live in the now. I can’t plan far ahead anymore, so I just appreciate what I can do today*” – Sharon .

This approach was a common thread among participants who coped better emotionally; they found that living “day-by-day,” rather than grieving an uncertain future, helped them manage anxiety and maintain hope. Participants redefined what made a day “worthwhile,” and actively reconstructed a sense of purpose within the confines of their mobility loss:

“*I’ve just learned to take each day as it comes. I still go out once a week to do my shopping when I’m feeling fine. I could do it online, but I don’t want to do that. I want to go out, I want my independence. Even if it’s just a short trip, it’s still important to me to keep doing something, to feel like I’m not giving up*” – Annie.

Additionally, some participants spoke about how living with a life-limiting illness often presented expectations from others to “live life to the full,” which was difficult when mobility had been impacted. Participants described how it was beneficial to reframe and focus on small positives:

“*I was often disappointed with myself and I gave myself a hard time when I felt that I was giving in. When you have a life limiting illness, there is a pressure on you not to waste time, just at a time when it is very difficult to live life to the full. But now, I’m quite happy lying on my bed and reading a book and I don’t want to feel that’s a bad thing*” – Lorraine.

Yet, adaptation was not purely individual. Caregivers too, found ways to restructure daily routines and offer support while protecting the autonomy of the person they cared for:

“*I try not to take over, but I do things in the background to help him [Charles] keep his independence. If I step in too much, it makes him feel worse*” – Patricia.

#### Evolving interdependence through mobility devices

Participants revealed that adapting to mobility loss was rarely a straightforward process of simply acquiring equipment. Instead, the use of assistive devices was often shaped by an ongoing negotiation between functional need, emotional readiness, and perceived social identity. Mobility devices were viewed by participants as both enabling and disabling. On one hand, they typically restored some access to daily life; on the other, they symbolised decline, dependency, and visible difference. Some participants embraced equipment early, often framing it as a practical and empowering tool that allowed them to retain a level of autonomy:

“*I realised quite early on that I would need a scooter if I wanted to go out, so I bought that quite early on. I don’t mind them, and I understand that they’re necessary*” – Philip.

Many described a “turning point,” a psychological shift where safety and autonomy outweighed concerns about visibility or pride. At that stage, equipment became a compromise: not a symbol of surrender, but a means of asserting control over one’s circumstances:

“*Yeah, now I couldn’t care less what people think. . . I’m much more receptive to equipment because the alternative is me falling. I suppose before, I had a bit of a sense of giving up if you use this piece of equipment*” – Claire.

Caregivers also played a key role in this process, often balancing their worries for their loved one to feel safe, whilst allowing them to retain some control:

“*It’s a fine line. I want her safe, but I also want her to feel like she’s still got some say in how she gets around*” – Matthew.

### Mobility loss: The unseen symptom

#### Missed opportunities for support

While mobility loss was deeply disruptive to participants’ everyday lives, it was rarely the focus of proactive discussion or intervention, leaving many people feeling unsupported and unseen by those providing their care, who could have referred them for therapy:

“*They [doctors] ask about my pain, ask how my appetite is, but no one asks about my walking. I don’t think it’s seen as important somehow*” – Kristen.

In some cases, participants reported that support was only introduced after their mobility had deteriorated, rather than as part of routine care. Even those who were housebound or reliant on mobility devices described minimal or delayed access to support. Several reflected that the opportunity for earlier intervention had passed them by, often with emotional consequences:

“*Once I was really struggling, the hospice stepped in, and they were brilliant, but I wish I’d had that kind of input before things got this bad*” – Charles.

Across interviews, participants described a healthcare system that was reactive rather than proactive in addressing mobility loss. Even when participants recognised their own functional losses, they felt unsure about where to turn or how to access help. Many described relying on personal initiative, informal advice, or online searches to source mobility devices or adapt their routines. This “do-it-yourself” approach highlighted a perceived absence of structured support:

“*Nobody seems to take any notice. I’ve just had to get on with myself. I bought my own walking stick, and Google, it’s a wonderful thing (laughs). I then looked up how to get it to my height*” – Elizabeth.

#### Stigma, ageism and illness assumptions

Participants described how mobility loss carried symbolic weight beyond its physical limitations, with mobility devices seen as more than functional aids. These devices were emotionally and socially charged, often viewed as visible markers of decline or associated with older age:

“*I haven’t used that [walker] in public. . . it’s a thing that old people use (laughs). I think it’s a pride thing about being seen using it*” – Kristen.

Such accounts reflected a blend of internalised, anticipated, and at times enacted stigma, where the use of equipment challenged how participants wished to be seen by others and themselves. Additionally, age and illness related assumptions further shaped how concerns were received in clinical encounters. Some participants felt their mobility loss was dismissed as a normal part of ageing or an inevitable aspect of advanced illness, rather than recognised as a symptom that could be supported:

“*When I mentioned my walking was getting worse, I was told, ‘Well you’re not getting any younger.’ But that’s not the point, I know my body, and this isn’t just age*” – Elizabeth.

## Discussion

### Summary of key findings

**Figure 1.**
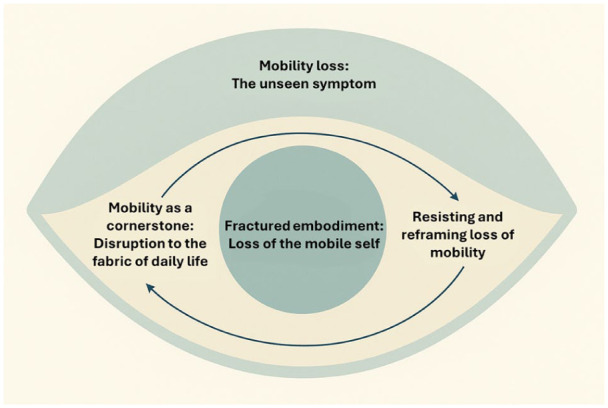
Visual representation of the four interconnected themes describing the experience of mobility loss in advanced cancer.

In our study, mobility loss in advanced cancer reshaped participants’ sense of identity, disrupted daily life, altered relationships, and prompted evolving coping responses. Four interconnected themes were identified (Figure 1), reflecting how the experience of mobility loss unfolded not only as a discrete physical symptom, but as a major reordering of self and social world.

This disruption reflects Bury’s concept of “Biographical Disruption,” where chronic illness interrupts the routines, identities, and expectations that give life coherence.^27^ Mobility loss eroded not only physical functioning but also valued roles, echoing studies showing that functional decline can impact self-esteem and social participation.^28,29^ As mobility declined, participants described their lives becoming smaller, routines more constrained and social contact more difficult. These experiences mirror the concept of “shrinking worlds” described in older adults with cancer,^30,31^ and Charmaz’s view of illness as an existential contraction.^32^

These changes also extended into relationships. As physical dependency increased, roles often shifted from partner to patient, or spouse to caregiver, introducing a loss of reciprocity. Such relational renegotiations have been observed in other caregiving dyads, where dependency intensifies emotional strain but can also foster new forms of interdependence.^33,34^ Alongside these social and relational shifts, mobility loss resulted in dissociation from the body. Some participants described their legs or hands as unreliable or unfamiliar, echoing Leder’s concept of “dys-appearance,” where the body becomes obtrusively present.^35^ Harris’s phenomenological work with people living with life-limiting illness similarly describes the body becoming an object of surveillance and struggle, especially when once-routine tasks like walking or dressing become difficult.^36^

In response to these disruptions, participants did not passively accept decline. In line with Morgan et al.′s concept of the “work of adaptation,” they actively engaged in emotional and practical strategies to preserve autonomy and continuity.^37^ These included simplifying routines, reframing expectations, and embracing mobility devices when they felt ready. Other studies similarly highlight how people with advanced cancer recalibrate what independence and normalcy mean, often redefining what counts as a “good day” or reshaping personal goals to maintain a sense of purpose.^38,39^ These emotion-focussed strategies, as described in Lazarus and Folkman’s transactional model of coping, may help sustain identity and ease distress in the face of illness.^40^

However, participants’ capacity to adapt was frequently challenged by systemic oversight. Despite mobility’s centrality to daily functioning and identity, it was rarely prioritised in clinical care. Participants described how consultations focussed on pain or appetite, while mobility was overlooked. Cheville et al. found that clinicians documented only 6% of the disabilities self-reported by people with cancer,^41^ while Pergolotti et al. reported that just 9% of older adults with cancer and mobility difficulties were referred for therapy.^42^ When functional loss is treated as an inevitable by-product of ageing or illness, or reduced to a single score, such as the ECOG Performance Status, the lived complexity of mobility may not be captured. This simplification contributes to missed opportunities for timely rehabilitation and support.^43^ As Carel argues, the medical gaze often overlooks aspects of illness that fall outside biomedical priorities, yet it is these neglected dimensions that profoundly shape lived experience.^26^

Even when willing to engage with support, participants faced barriers rooted in stigma and ageist assumptions. For many, mobility devices signalled failure or loss of dignity. This echoes research showing assistive devices are often viewed as both enabling and exposing.^44,45^ Wendell’s theory of “The Social Construction of Disability” offers a lens for understanding this dynamic.^46^ She reframes disability not as an individual deficit, but as a socially-mediated experience shaped by norms that valorise independence, youth, and physical capacity.^46^ Interventions must therefore address not only physical function, but also the symbolic dimensions of mobility loss.

### Clinical implications and future directions

Routine mobility assessments and timely referrals to rehabilitation should be embedded within oncology and palliative care to support both function and quality of life.^47,48^ Participants’ reliance on self-sourced mobility devices highlights gaps in anticipatory support. Whilst some secured suitable equipment, others risked using inappropriate devices due to limited guidance. Without professional input, individuals may select devices that are inappropriate, contributing to underuse or abandonment.^49^ Mobility-specific advance care planning tools could prompt timely, person-centred conversations and ensure support remains aligned with evolving needs.^50^ Future research should examine how cultural values influence experiences of mobility and its interaction with other symptoms. It should also explore whether timely mobility support can reduce hospitalisations, emergency admissions, and caregiver strain.

Whilst this study was conducted in England, the findings hold significant relevance for international oncology and palliative care communities. Global frameworks, such as those from the World Health Organisation, emphasise mobility and social participation as essential components of quality of life.^51^ This underscores the importance of proactively addressing mobility loss as a clinical priority across diverse healthcare settings. Moreover, there is growing international consensus on the need to integrate rehabilitation earlier in the cancer care continuum, supported by function-based assessments rather than sole reliance on global performance scores.^52^ These findings contribute to this evolving discourse and may inform practice and policy in both high-resource and resource-limited settings.

### Strengths and limitations

A key strength of this study is its longitudinal design, using two interviews to capture evolving experiences. The research team’s combination of clinical and non-clinical backgrounds strengthened the interpretative process by enabling sensitivity to both embodied experience and contextual nuance. This was supported by ongoing reflexivity and regular team discussions to ensure interpretations remained grounded in participants’ accounts. The inclusion of caregivers provided some insight into experiences and relational dynamics, but the small number limits the depth and breadth of caregiver perspectives. As such, findings are primarily interpreted through the lens of patients’ accounts. To expand on this, a supplementary workshop was held with three bereaved caregivers from minoritised ethnic backgrounds. While the themes largely resonated, participants in the workshop noted a greater emphasis on collective and family-based care in their communities, suggesting caregiving dynamics not fully captured in the study.

## Conclusion

Mobility loss in advanced cancer is not only a physical symptom, but a personal and relational disruption. As mobility decreases, patients and families navigate changes in identity, participation, and roles, often accompanied by emotional and social consequences. Recognising mobility as a core clinical concern can help ensure that care addresses not only physical function but also the broader psychological and social impact of advanced illness.

## Supplemental Material

sj-docx-1-pmj-10.1177_02692163251400115 – Supplemental material for The unseen symptom: A longitudinal qualitative interview study exploring mobility loss in people with advanced cancer Supplemental material, sj-docx-1-pmj-10.1177_02692163251400115 for The unseen symptom: A longitudinal qualitative interview study exploring mobility loss in people with advanced cancer by Carmine Petrasso, Lisa Jane Brighton, Matthew Maddocks and Joanne Bayly in Palliative Medicine

sj-docx-2-pmj-10.1177_02692163251400115 – Supplemental material for The unseen symptom: A longitudinal qualitative interview study exploring mobility loss in people with advanced cancer Supplemental material, sj-docx-2-pmj-10.1177_02692163251400115 for The unseen symptom: A longitudinal qualitative interview study exploring mobility loss in people with advanced cancer by Carmine Petrasso, Lisa Jane Brighton, Matthew Maddocks and Joanne Bayly in Palliative Medicine
