# Supplementary material for: The unseen symptom: A longitudinal qualitative interview study exploring mobility loss in people with advanced cancer
Source: Palliat Med. 2025 Dec 26;40(2):206–16. doi: 10.1177/02692163251400115 (PMC12852488; doi:10.1177/02692163251400115)
Supplement: sj-docx-1-pmj-10.1177_02692163251400115 – Supplemental material for The unseen symptom: A longitudinal qualitative interview study exploring mobility loss in people with advanced cancer [file sj-docx-1-pmj-10.1177_02692163251400115.docx]

**Participant Topic Guide**

1. **Introduction:**
   - Welcome and obtain informed verbal consent from both the patient and/or carer.
   - If you need to stop, or do not wish to answer a question, please just let me know.
   - Have you got any questions before we start?
2. **Background Information:**

- Opening discussion; finding out about the participant/ carer. Tell me a little about what made you interested in this research project?

1. **Understanding Mobility Changes:**
   - Thank you for that, and the first area I’d like to explore is understanding any changes in your walking ability.
   - Potential areas of focus for patient:
     - Has your walking changed since your diagnosis of cancer? What changes have occurred? How have these impacted on your ability to complete your usual daily activities such as washing, dressing etc.?
     - Have changes in your walking impacted your ability to be independent and/or prevented you from participating in social activities?
     - How has a change in your walking affected your quality of life?
     - Has a change in walking impacted on your future goals/ plans?
   - Potential areas of focus for carer:
     - From your perspective, how have you observed changes in (person’s name) walking?
     - Have these changes affected their independence or social activities?
2. **Mobility Interventions:**
   - Now what I’d like to move onto, is better understanding any potential interventions you may have received that has helped your walking:
     - For example have you been offered or participated in any programmes (non-pharmacological) since being diagnosed with cancer?
     - If yes, describe the programme and its impact.
     - If no, would you consider participating in such a programme?

1. **Assistive Devices:**
   - The next area is looking at assistive devices, this is any piece of equipment that helps you with your walking
     - And so have you started using any assistive devices since your cancer diagnosis?
     - If yes, what prompted this decision, and how has it affected your daily life? Was the assistive device recommended by a professional?
     - If no, have you been advised to use any assistive devices, and would you consider using them?
     - Have you noticed any changes in (person’s name) willingness or reluctance to use assistive devices?
     - How do you feel about the use of assistive devices in terms of supporting (person’s name)?
2. **Emotional and Social Impact (Adaptation and Coping Strategies):**
   - So the penultimate area for discussion is considering any coping strategies or mechanisms you have developed.
   - Has a change in walking impacted on your mental health?
   - Potential questions may include:
     - So have you developed any strategies to cope with the challenges posed by changes in walking?
     - Have you received support or assistance from family, friends, or support networks? If so, what sort of support. What has been the most helpful.
3. **Recommendations:**
   - Do you have any recommendations or suggestions for healthcare professionals, other caregivers, or researchers regarding optimising walking in people with cancer?
4. **Conclusion:**
   - Before I end the recording, have you got any comments, or anything further you would like to add about the impact cancer has had on your walking?
   - Thank them for their participation in the interview.
