# Supplementary material for: The unseen symptom: A longitudinal qualitative interview study exploring mobility loss in people with advanced cancer
Source: Palliat Med. 2025 Dec 26;40(2):206–16. doi: 10.1177/02692163251400115 (PMC12852488; doi:10.1177/02692163251400115)
Supplement: sj-docx-2-pmj-10.1177_02692163251400115 – Supplemental material for The unseen symptom: A longitudinal qualitative interview study exploring mobility loss in people with advanced cancer [file sj-docx-2-pmj-10.1177_02692163251400115.docx]

## Supplementary file: Biographies (pseudo-anonymised)

**Charles:**

Charles, a gentleman in his late 70s lives at home alongside his wife Patricia. Before his diagnosis, Charles was a keen walker, enjoying mountain walks and other physical activities. His renal cell carcinoma diagnosis resulted in a gradual decline in his walking abilities, but it was only after radiotherapy to his hip, that his mobility rapidly declined. During the first interview, he described a time when he had become completely bedbound and dependent. However, Charles remained optimistic and soon regained the ability to walk outside with a crutch. He continued to display a level of resilience and acceptance and focused on his incremental improvements.

Charles explained that despite the challenges he faced, he was motivated to try and regain more mobility, with goals such as being able to walk around his home and use the facilities independently. Unfortunately, the second interview occurred following a decline in his health, and resulted in him needing to use assistive devices, such as a frame. He had also started working with a physiotherapist privately as well as the hospice physiotherapist to improve his strength. By the second interview, Charles’ quality of life had been dramatically affected by his mobility decline, particularly in social activities and his ability to participate in things he once enjoyed, like walking around the city and going to restaurants. He missed the sense of accomplishment from working and contributing but still enjoyed engaging with friends and family through email and still tried to have a positive outlook. He was concerned about the ongoing impact his loss of mobility would have on his wife Patricia and wanted to ensure she was looked after too.

**Patricia**

Patricia is Charles’ wife and primary carer. She is in her mid-70s and balances the demands of caregiving with her own health challenges. A former nurse, Patricia felt well-equipped to handle the medical aspects of Charles' care, but found the physical demands of his immobility difficult to manage. She describes the experience as challenging, particularly in terms of physically supporting Charles as he transitioned from being somewhat mobile to requiring constant assistance. The shift from him being independent to needing her for tasks like moving from one room to another or using the commode was particularly difficult for her and she had to quickly adapt to her new responsibilities.

Patricia's perspective on caregiving is pragmatic. She had become adept at managing Charles' medical needs, including organising pain relief and coordinating with hospice care. She also adjusted to the sudden shift in their lifestyle. Once enjoying a more social life, including activities like attending the city club and having friends over, she now spends much of her time ensuring Charles’ needs are met. She also faced emotional challenges, particularly with the strain of managing Charles’ care and the toll of watching his decline. Despite this, she too remained optimistic and found comfort in the relationships built with hospice and care agency staff and was very appreciative of their support.

**Kristen:**

Kristen, in her mid-60s, has always been an active person, working as a teacher and enjoying outdoor activities like walking her dogs. However, since being diagnosed with metastatic breast cancer, her mobility significantly declined due to side effects from treatments, such as tendon damage and joint instability. Initially, Kristen’s experience with oncology care was negative, as she felt that medical professionals were primarily focused on managing her cancer and were unaware of, or dismissive towards, the side effects she was experiencing.

Kristen describes how simple tasks, like walking her larger dog or carrying shopping, had become impossible, and even moving around her own home had become a challenge. Kristen expressed a sense of frustration of having to plan every movement and the loss of spontaneity in her activities resulted in significant emotional hurdles for her. Despite this, she demonstrated resilience by actively seeking solutions and striving to remain as mobile as possible.

In the second interview, Kristen reported some improvement in her walking, especially on flat surfaces. She no longer relied on a walker but still used a walking stick for extra support when navigating stairs or uneven terrain. She remained hopeful, although realistic, about her mobility recovery.

Kristen’s experience had also been shaped by her role as a caregiver to her disabled husband, and the couple’s shared mobility issues further complicated their plans for the future. However, she remained determined to stay active and maintain some level of independence, using mobility aids like a walking stick and adjusting her environment to accommodate her new limitations. Kristen also expressed the need for better early interventions and rehabilitation to support people with cancer in maintaining their mobility, highlighting the lack of coordinated care and the burden of self-advocacy in navigating the healthcare system.

**Daniel:**

Daniel is a gentleman in his late 70’s who, before his diagnosis, had led an active lifestyle, enjoying activities like skiing and mountain biking. However, since his diagnosis of multiple myeloma, his health drastically declined. The chemical treatments, including chemotherapy and radiation, left him with severe mobility issues. The cancer spread to his spine, causing fractures that caused him pain on movement.

Daniel described a lack of control and feeling of instability made even simple tasks, such as walking to the shop or navigating stairs, extremely difficult. His ability to walk long distances declined, and he relied on mobility aids such as walking poles and a rollator, though he found these both limiting and difficult to use in certain environments.

Despite these challenges, Daniel was determined to push through and maintain some level of independence. He aimed to walk 3,000-4,000 steps per day, but explained how his reduced mobility limited his social life and ability to participate in activities.

Daniel's emotional health has also been deeply impacted by his physical decline. He expressed feelings of anger and frustration, especially with his initial misdiagnosis and the resulting damage to his spine. This grief is compounded by the loss of independence, the difficulty of relying on others, and the emotional toll of watching his family sacrifice their own lives to care for him. However, Daniel was trying to find small moments of normalcy, like using his walker to go out for a short time with friends or family, though he often felt left out of conversations about activities he could no longer participate in.

Daniel continued to receive support from various healthcare services, including hospice care and physiotherapy, though he felt the system has not provided enough proactive intervention, particularly with regards to mobility rehabilitation.

**Elizabeth:**

Elizabeth, a lady in her 80s, lives alone having lost her husband to cancer several years ago. As a result, she became increasingly involved within her community. Elizabeth’s was diagnosed with colon cancer, which later spread to her lungs. Despite the challenges associated with her cancer, Elizabeth remained a resilient and practical person, always looking for solutions to her mobility issues.

Prior to her cancer diagnosis, Elizabeth lived an active life, enjoying social events, gardening, and travel. However, as her health deteriorated, particularly with the onset of severe pain in her hip, her mobility became increasingly restricted. In the early stages of her diagnosis, Elizabeth found it difficult to get answers from her healthcare providers regarding her walking issues, with many dismissing it as a result of her age. This lack of recognition of her walking difficulties frustrated her.

Elizabeth reflected on how previously simple tasks like going to the shops or visiting friends became more difficult. The act of walking became a source of frustration, particularly the inability to take the first step when rising. Elizabeth’s situation improved slightly after receiving a steroid injection into her hip, which relieved some of her walking difficulties. While she still couldn’t walk long distances, Elizabeth felt grateful for the improvement and the renewed confidence it had given her to engage in activities she had previously avoided.

**Sharon:**

Sharon is a 50-year-old woman with a diagnosis of metastatic breast cancer. Prior to her diagnosis, she had a fulfilling career as an office manager, loved gardening, and enjoyed a high level of physical activity. However, as her cancer progressed and she began her treatment regime, Sharon's mobility was significantly impacted. She developed pain in her hip and back, which made even simple tasks such as walking difficult. Sharon relied on a walking stick and, more recently, crutches provided by her physiotherapist.

Her physical limitations not only affected her daily life but also her quality of life. Gardening, which she once loved, had become an exhausting task, and she could no longer engage in spontaneous activities like she did before; "I used to love gardening, but I can’t, I just can’t do it now. I can’t get down there, I can’t get back up. It’s just exhausting". This loss of independence effected other activities like food shopping, which now requires careful planning and assistance.

Sharon's coping strategies focussed heavily on maintaining her independence, despite the constraints of her physical limitations. Sharon was committed to staying as active as possible and enjoyed participating at the local 'Living Well' centre. These activities, along with regular treatments like acupuncture and reflexology, provided Sharon with both physical relief and mental clarity.

Despite the changes in her lifestyle, Sharon continued to find ways to navigate her new reality. She still drove when she felt able, took trips to the supermarket, and met with friends for coffee.

**Matthew:**

Matthew, in his late 70s, is Claire’s primary carer, a role he has taken on since her cancer diagnosis. Before Claire’s diagnosis, Matthew and Claire led a highly active lifestyle, enjoying frequent travel and outdoor activities. However, since her diagnosis, Matthew’s life has shifted dramatically. Initially, he was not very engaged in caregiving duties, as Claire managed most aspects of their life, but over the years, the increasing severity of Claire’s illness has meant that Matthew has assumed a greater caregiving role. This transition has been challenging for him, particularly as Claire’s health has worsened. He soon found himself managing all aspects of their daily lives, from Claire’s medication and medical appointments to ensuring her physical safety.

Matthew’s own health has also been affected by the stresses of caregiving. Despite these challenges, he continues to care for Claire, though he admitted that this is becoming increasingly challenging. He is particularly concerned about the lack of coordinated support for caregivers like him, feeling that the healthcare system failed to provide the kind of proactive, ongoing support they need. The absence of a dedicated team to guide them through their cancer journey made Matthew feel isolated and unsupported, even though he recognises that the care Claire receives from the hospice and cancer unit is very good.

As a caregiver, Matthew had to come to terms with the overwhelming sense of loss. He noted that their once vibrant social life had diminished significantly, and they no longer felt able to travel or engage in social outings. The uncertainty of Claire’s health and the constant reorganisation of their schedules around her treatments left Matthew with little room to focus on his own needs. The couple's once joyful and active life has been replaced with routine hospital visits, treatment schedules, and increasing uncertainty about the future.

**Claire:**

Claire is in her late 70s and diagnosed with bowel cancer, which had metastasised. Prior to her diagnosis, Claire led a highly active and independent life, filled with travel, socialising, and engaging in various activities. She and her husband, Matthew, were keen travellers, frequently taking cruises and enjoying outdoor activities. Claire’s diagnosis, however, resulted in her transitioning from an active lifestyle to one limited by her illness.

Claire described the frustration of losing her previous freedom and independence. Her mobility challenges, including pain and weakness, have restricted many of her favourite activities. While she continues to face these challenges, Claire has sought new ways to adapt and maintain a sense of purpose. She turned to art, attending multiple art classes each week, a pursuit she now uses to stay mentally engaged and socially connected.

Although Claire's mobility showed some signs of improving, she experienced physical limitations, and the struggle between her desire for independence and the reality of her condition remains a constant battle. Claire expressed difficulty in acknowledging her progress, explaining how she did not see the improvements.

**Annie:**

Annie is a 60-year-old lady, diagnosed with metastatic breast cancer. She originally lived abroad with her husband and had been active, leading a busy life with a mix of work and personal interests. Annie described how her cancer led to lots of physical challenges, particularly regarding her walking.

Annie experienced both physical and emotional shifts, especially concerning her mobility. She previously could walk without much thought but had to then rely on a walking stick to support her when out and about. She also used a frame to assist her with daily tasks such as getting in and out of bed and described feeling more dependent on her husband for physical support. By the second interview, Annie’s anxiety about her mobility increased, and she found herself thinking twice before attempting tasks or leaving the house. Despite this, she was determined to continue engaging in the activities that were still possible for her.

The changes in her physical abilities have impacted her relationships, and she expressed frustration of not being able to care for her grandchild in the way she once could.

Annie’s coping strategies involved staying engaged with the people around her, particularly through her participation in the "Living Well" programme and maintaining close connections with her friends and family. She also tried to maintain a sense of normalcy by keeping a diary of her daily activities and adjusting her lifestyle to manage her energy levels. However, she still grappled with feelings of guilt and frustration, particularly when she was unable to contribute in the ways she once did. Despite the challenges, Annie remained hopeful. She continued to engage with rehabilitation options and was open to exploring further mobility devices if they would help her regain some independence.

**Peter:**

Peter, in his early 70s, was diagnosed with metastatic prostate cancer. Over the years, Peter has undergone various treatments, including hormone injections, radiotherapy, and chemotherapy, which have had significant effects on his mobility. Despite his illness, Peter remained positive and proactive about his condition. Peter explained how his mobility gradually declined, especially after his chemotherapy treatments, which left him with significant pain and weakness in his lower limbs. However, Peter maintained his independence, engaging in Tai Chi to manage his balance and mobility, which he attributed to helping him regain strength in his legs and improve his overall balance.

Peter's physical health has been an ongoing struggle, with persistent aches and pains that have made even simple tasks more difficult. He described his walking as awkward, often compared to someone who has had polio, with a distinctive "clip-clop" gait that is both painful and frustrating. His part-time work was important to him and provided him with a sense of purpose.

Peter remained fiercely independent, resisting mobility devices such as walking sticks and stairlifts, which he felt would signify a loss of autonomy. Peter remained generally upbeat, though he was aware of the limitations his reduced mobility may have on him in the future.

**Philip:**

Philip, a 72-year-old man, had enjoyed an active life and fulfilling career. However, Philip's life drastically changed following his oesophageal cancer diagnosis.

Before his diagnosis, Philip lived independently in a motor home and enjoyed hobbies like narrow boating and visiting his friends. However, the cancer quickly impacted his mobility, leaving him unable to engage in activities he once enjoyed, describing how his walking had become difficult and how every aspect of his life had been affected. He explained the difficulty in even simple tasks, like getting in and out of bed, that once seemed easy.

Philip’s transition from living independently to moving in with his family was difficult. While he was grateful for their support, he felt like a burden and despite the love for his family, he struggled with his loss of autonomy. Philip’s use of mobility aids, including a walking frame and mobility scooter, had been a necessary adjustment. He acknowledged their importance but also saw them as symbols of his gradual decline and suggesting that while the devices provided support, they did not solve the underlying issue of his deteriorating health.

Despite these challenges, Philip tried to stay connected with others. He used his mobility scooter to visit the café and talk to friends, though even this simple activity had become more difficult due to his mobility limitations. He also acknowledged the limitations that came with it, including the frustration when the scooter malfunctioned. Philip’s mental and emotional well-being had been deeply affected by his physical decline. He reflected on the pain and loss of control, particularly the impact on his ability to care for himself and emphasised how this loss had been one of the hardest aspects of his illness.

**Lorraine:**

Lorraine, in her mid-60s, has faced significant challenges since her breast cancer diagnosis. After several recurrences and treatments, her cancer metastasised to her bones, particularly affecting her spine and feet, which majorly impacted her mobility. Lorraine had undergone numerous treatments, including chemotherapy, immunotherapy, and bisphosphonates, all of which have contributed to bone fractures and ongoing pain. Despite these challenges, Lorraine remained proactive in managing her condition and maintaining her mobility as much as possible.

Lorraine developed strategies to cope with her mobility limitations, such as using appropriate footwear, engaging in physiotherapy, and maintaining a regular walking routine. Lorraine also uses an Oura ring to track her sleep and activity, which helped her adjust her routine.

Lorraine’s self-awareness increased over time, with an improved understanding of how her body responded to movement and rest. However, her mobility challenges led to a decline in her confidence. She often faced anxiety about her physical limitations, especially when engaging in activities that required her to be out of the house for extended periods. Lorraine expressed how the combination of physical pain, fatigue, and the psychological toll of cancer had restricted her social life, making her more isolated. Despite this, Lorraine remained committed to staying connected with her family and friends, and she continued to challenge herself to participate in life as much as she could, even if it required adaptations and careful planning.
